# The Redder the Better: Wing Color Predicts Flight Performance in Monarch Butterflies

**DOI:** 10.1371/journal.pone.0041323

**Published:** 2012-07-25

**Authors:** Andrew K. Davis, Jean Chi, Catherine Bradley, Sonia Altizer

**Affiliations:** Odum School of Ecology, The University of Georgia, Athens, Georgia, United States of America; National Tsing Hua University, Taiwan

## Abstract

The distinctive orange and black wings of monarchs (*Danaus plexippus*) have long been known to advertise their bitter taste and toxicity to potential predators. Recent work also showed that both the orange and black coloration of this species can vary in response to individual-level and environmental factors. Here we examine the relationship between wing color and flight performance in captive-reared monarchs using a tethered flight mill apparatus to quantify butterfly flight speed, duration and distance. In three different experiments (totaling 121 individuals) we used image analysis to measure body size and four wing traits among newly-emerged butterflies prior to flight trials: wing area, aspect ratio (length/width), melanism, and orange hue. Results showed that monarchs with darker orange (approaching red) wings flew longer distances than those with lighter orange wings in analyses that controlled for sex and other morphometric traits. This finding is consistent with past work showing that among wild monarchs, those sampled during the fall migration are darker in hue (redder) than non-migratory monarchs. Together, these results suggest that pigment deposition onto wing scales during metamorphosis could be linked with traits that influence flight, such as thorax muscle size, energy storage or metabolism. Our results reinforce an association between wing color and flight performance in insects that is suggested by past studies of wing melansim and seasonal polyphenism, and provide an important starting point for work focused on mechanistic links between insect movement and color.

## Introduction

Monarch butterflies (*Danaus plexippus*) are among the world’s best-known insect species, partly because of their spectacular two-way bird-like migration in eastern North America [1], and also because of their aposematic orange and black wing coloration, which advertises their bitter taste and toxicity to potential predators [2]. Variation among individuals in both orange hue and the extent of black wing pigmentation can be subtle and difficult to discern with the naked eye, but can vary in biologically meaningful ways. For example, one study demonstrated that subtle variations in the amount and intensity of black pigment on wings (i.e. wing melanism) depended on both larval rearing temperature and monarch population origin [3]. Other work showed that the saturation of orange on male monarchs’ wings predicted their future mating success [4] and the wings of fall migrating monarchs in eastern North America expressed a darker shade of orange (i.e. more red) relative to the wing color of non-migrating summer cohorts [5]. The observation of redder wing color in migratory monarchs suggests a possible link between wing color and flight propensity in this species.

Prior studies of wing coloration and flight behavior in butterflies show that these traits are often related. In species with seasonal color morphs (i.e., seasonal polyphenism), comparison between generations shows differences in wing traits that could affect flight ability between seasonally-varying thermal environments [6]. For example, Kingsolver [7] showed that for the seasonally polyphenic butterfly *Pontia occidentalis*, selection favored darker hindwings during the spring generation that experiences cooler temperatures, and paler hindwings during the summer generation that experiences hotter temperatures. Other work examining butterflies at high elevations found that wing melanism is positively associated with the amount of time spent in flight at low temperatures [8], [9]. Similarly, in the speckled wood butterfly (*Pararge aegeria*), darker males spend more time in flight than paler males, most likely because of their increased warming rates [10].

In this study we used a tethered flight mill apparatus to quantify the flight distance and speed of captive-reared monarchs relative to wing color variation. Specifically, we examined data from three separate experiments where monarchs were sampled shortly after eclosion to measure wing traits (color and morphology) and were later used in flight trials. Based on past work showing redder wing hue in fall migratory monarchs relative to non-migratory summer cohorts [5], we expected that individuals with redder wing coloration might show greater measures of flight speed or distance. We also examined whether wing melanism (based on the total area of black pigmentation) correlated positively with flight speed, as might be expected from past work on wing melanism and flight behavior in butterflies [7], [8], [9], [10].

## Materials and Methods

### Ethics Statement

All necessary permits were obtained for the collection and transport of live monarch butterflies (see below). No permits were necessary for the experimental work, and this project did not involve endangered or protected species.

### Butterflies

Butterflies used in all three experiments were the non-inbred progeny and grand-progeny of wild-captured monarchs from the large migratory population in eastern N. America. Experiment 1 was conducted using the direct progeny of crosses between wild adults captured from Atlanta, GA, USA and Ithaca, NY, USA between Aug-Sep 2003. Experiment 2 monarchs were the progeny of adults captured at one site in Atlanta, GA, USA. Wild adults were collected using aerial nets from public roadsides that were not privately-owned or protected (collecting non-endangered butterfly species in non-protected public areas does not require a permit in the USA). All butterflies were transported to our lab (at Emory University, Atlanta, GA for Experiments 1 and 2) under permission from the United States Department of Agriculture (USDA PPQ-526 Permit # 54756). Monarchs used in Experiment 3 were the grand-progeny of wild-caught adults captured in Feb 2008 (using butterfly nets on extendable poles) at two wintering sites in Michoacan, Mexico. This set of monarchs was collected with permission from SEMARNAT (Secretaria de Medio Ambiente y Recursos Naturales, Permit # 08202) and the Monarch Biosphere Reserve (Mariposa Monarcha Reserva de la Biosfera, Permit # RBMM-DIRECT-0050.08), then transported to our laboratory at the University of Georgia (Athens, GA USA) with permission from PROFEPA (Procuraduría Federal de Protección al Ambiente, Permit # 23811 21/Enero/2008), the U.S. Fish and Wildlife Service (Customs Document # P526P-06-02137) and the USDA (Permit # P526P-06-02137).

All captive-raised monarchs were fed cuttings of greenhouse-raised swamp milkweed (*Asclepias incarnata*) in plastic containers (4.7 L containers at densities of 5 larvae per container in Experiments 1 and 2, and 0.47 L with one larva per container in Experiment 3). During rearing, monarch larvae were exposed to natural light conditions from large windows facing southeast. Larvae for each of the three experiments were reared in late-summer, so that they were exposed to natural day lengths for the time of year immediately prior to the monarchs’ fall migration. Upon eclosion, adults were checked for infections with the protozoan parasite, *Ophryocystis elektroscirrha* [11]. Only non-infected butterflies were considered here since infections are known to reduce flight performance [12]. Thereafter monarchs were stored in glassine envelopes in an incubator (set to 13°C) until measurement (below).

### Morphometric and Wing Color Data

Between 1 and 3 days post-eclosion, we scanned the entire dorsal side of each monarch (including wings) with a digital flatbed scanner, following Davis et al. [3], [4]. Because a separate scanner was used for Experiment 3 (which resulted in slightly different average wing hue scores), we included experiment as a blocking variable in the analyses below. During scanning, we made certain that the resulting images were not altered by the scanning software (i.e. all image-enhancing options were turned off) to ensure consistent color-measurement. After scanning, butterflies were returned to the incubator (in envelopes) until flight trials. With the scanned images, we used the FoveaPro plugin software (Reindeer Graphics, Inc.) for Adobe Photoshop®, to measure the area (in mm^2^), length and width (in mm) of right and left forewings. From these values we calculated the wing aspect ratio (L/W), which is a predictor of migratory distance among monarch populations [13] and therefore could impact individual flight performance. We also measured the two-dimensional body area (combined head, thorax and abdomen, in mm^2^) and divided this by forewing area to derive a body-to-wing-size parameter as a proxy for wing loading [13].

To quantify wing color, we calculated the proportion of black pigmentation on each forewing (an index of melanism) following Davis et al. [3]. We next measured the orange hue by obtaining the average pixel hue score of the orange portion of the CuA_1_ cell from each forewing [4], [5] (File S1). With digital images, hue is measured in degrees around a 360° circle, with 0° being perfect red, and each pixel in an image has a corresponding hue score. At the scanner settings we used (300 dpi), the section of the wing where we measured hue typically contained between 7000–9000 pixels. This method of estimating color differs from other studies where spectrometers are used to measure wing reflectance spectra [14], [15], [16]. Because of this, we compared hue data obtained from the scanner method with reflectance data obtained from a USB2000 miniature fiber optic reflectance spectrophotometer for a different set of recently-emerged captive-reared monarchs. Wing hue scores measured from digital scans were significantly correlated with reflectance measures between 480–780 nm (in the visible range of orange-red; File S1).

### Flight Trials

**Figure 1:**
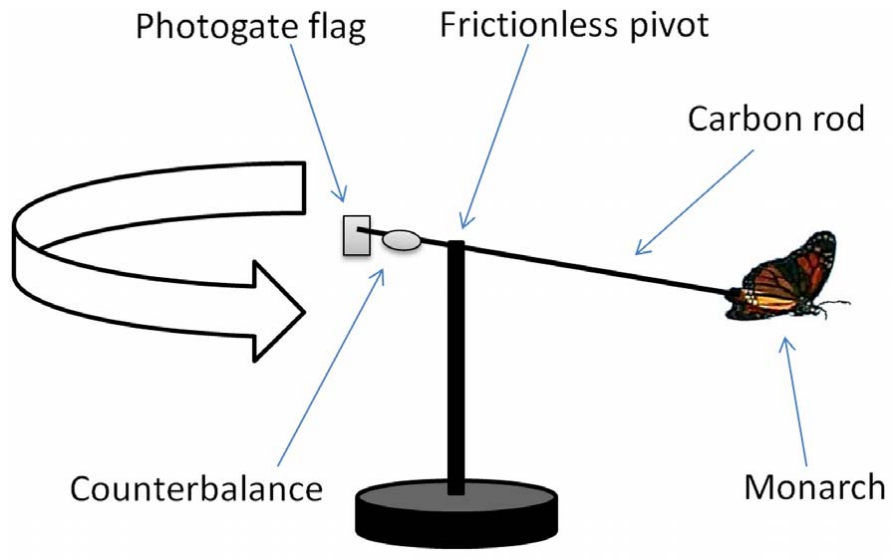
Schematic of butterfly flight mill used in all experiments. A lightweight rod was attached to a post via a near-frictionless pivot. A thin wire was glued to the thorax of each monarch and the wire was attached to the rod during flight trials. A 5 cm flag was attached to the other end of the rod, and the flag passed through a photogate (not shown) with each rotation so that the speed and duration of flight could be monitored via computer. A video of this apparatus is provided as supplemental material (Video S1).

The flight performance of monarchs in all three experiments was tested using a tethered flight mill described previously [12]. Briefly, the apparatus was composed of a lightweight carbon rod 120 cm in length and 3 mm in diameter, threaded through a stainless steel pivot to provide a near-frictionless rotation, and with a movable counterbalance to account for variation in each monarch’s weight (Fig. 1). An infrared beam emitted by a photogate was interrupted by a flag attached to one end of the carbon rod to record the time elapsed during each rotation. Butterflies were attached to one end of the rod using an ultralight steel wire glued to the thorax. Immediately following release, >90% of individuals initiated flight (see Video S1) and those that did not were removed from the study. For each flight trial we recorded the total time (in min), total distance (in km) and average speed (total distance divided by total time). A flight was terminated when the monarch remained still (no wing movement and rotations completely halted) for more than ten full seconds, and each individual was used only once in the data set. All monarch rearing and flight mill experiments were performed in laboratory facilities at Emory University (Atlanta, GA) and the University of Georgia (Athens, GA). The flight mill trials were performed in an indoor room where temperature remained constant throughout the experiments. No specific permits were required for the flight experiments, nor did this project involve endangered or protected species.

### Data Analyses

Measures of wing size, shape and color were averaged between left and right forewings prior to analysis. To test for possible associations between flight measures and wing color, we used general linear modeling, where total flight distance (log-transformed), total flight duration (square root transformed) and flight speed (km/hr) were separate response variables, sex and experiment were categorical predictors, and wing area, aspect ratio, relative body size, orange hue score and percent black were continuous covariates. Analyses were conducted using Statistica 6.1 software [17].

## Results

**Figure 2:**
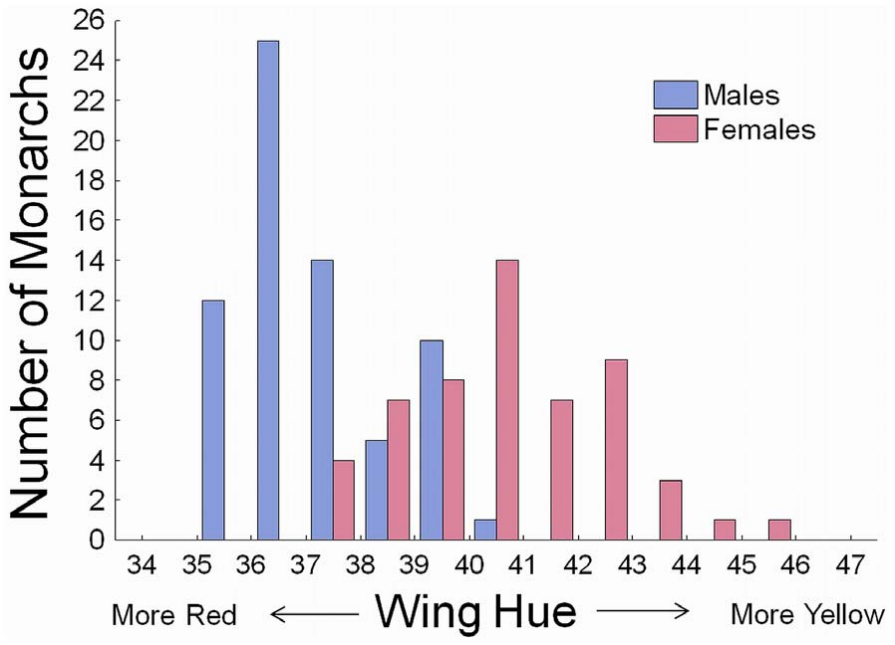
Frequency-distribution of male and female orange hue scores aggregated across experiments 1–3. See methods for description of color measurement.

A total of 121 monarchs were flown across all three experiments. The average flight distance was 4.0 km (±3.1SD, min 0.1, max 13.4), the average flight duration was 61.3 min (±45.1SD, min 2.6, max 143.2) and average flight speed was 3.9 km/hr (± 1.1SD, min 1.4, max 8.5). The wing hue scores varied along a continuum from 35.9 to 45.7 degrees, with males tending to have lower hues (redder wings) than females (Fig. 2).

**Figure 3:**
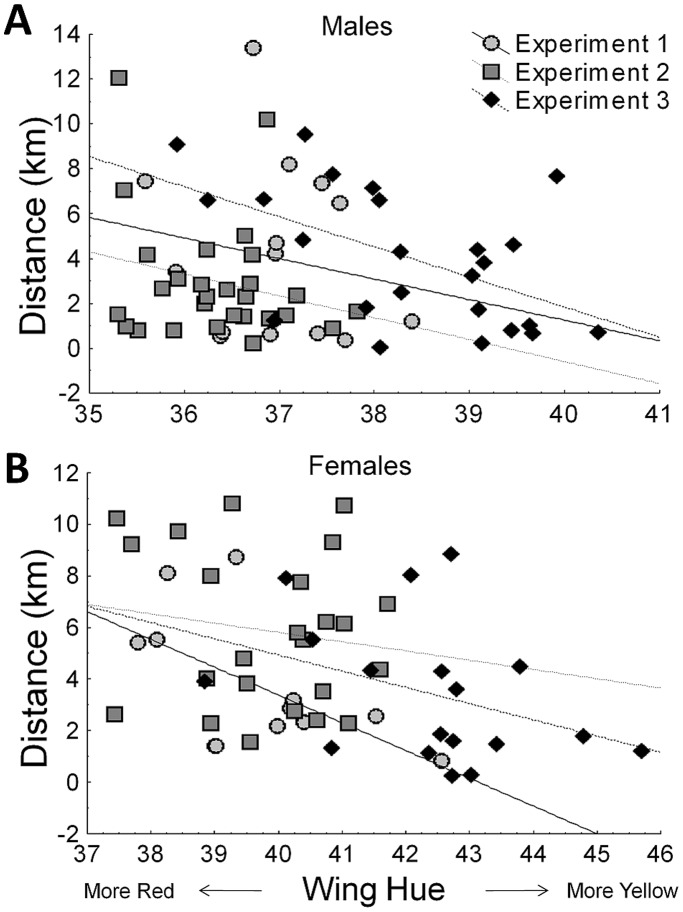
Relationship between orange hue score and distance flown on the flight mill for all male (A) and female (B) monarchs in this study. Note that the x- and y-axis scales differ for the two sexes.

Females flew farther distances on average than males (4.57 km ±3.01SD vs. 3.59 km ±3.11SD) and this effect of sex on flight distance was significant (F_1,112_ = 7.059, p = 0.009). Moreover, there was a negative relationship between orange hue and flight distance (F_1,112_ = 9.785, p = 0.002), such that individuals with lower hue scores (redder wings) travelled farther on the flight mill within each sex (Fig. 3). Of the remaining variables examined, there was no significant effect of forewing area (F_1,112_ = 0.331, p = 0.566), wing aspect ratio (F_1,112_ = 0.425, p = 0.519), relative body size (F_1,112_ = 1.086, p = 0.299), percent black (F_1,112_ = 1.179, p = 0.280) or experiment number (F_2,112_ = 1.681, p = 0.191) on flight distance.

**Figure 4:**
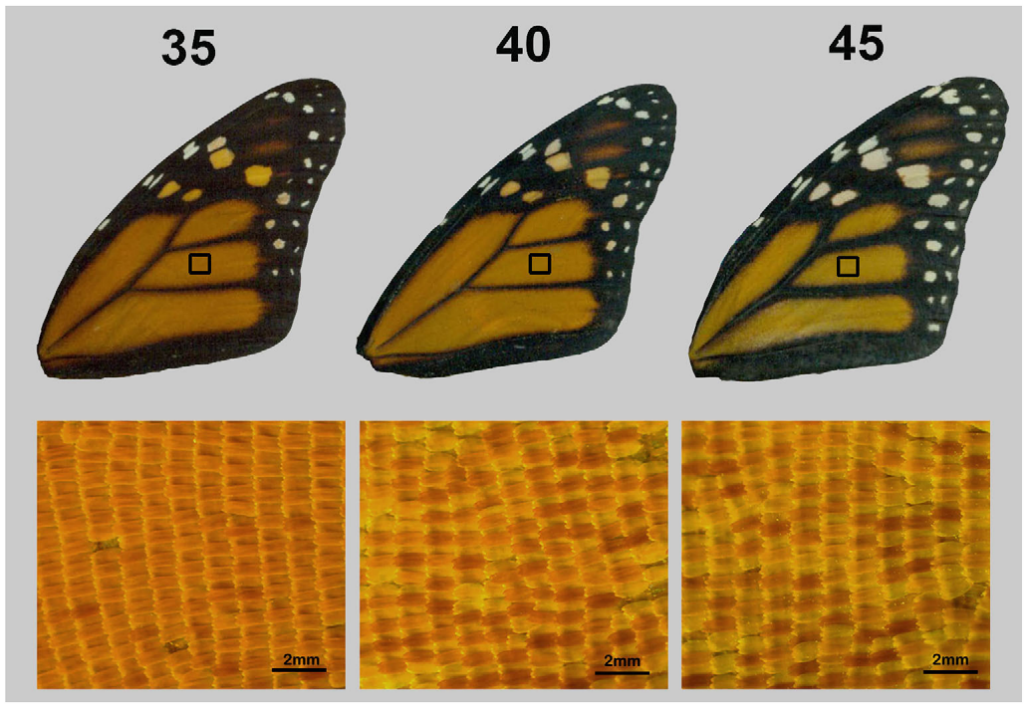
Forewings from three monarchs that span the range of hue scores (values above wings) in this study. Magnified sections show close-up of scales from each individual.

Analysis of flight duration (time in hr) using the same predictor variables were virtually identical to results for flight distance (File S2, Table S1), which is not surprising as we found a strong positive correlation between these two response variables (Pearson’s R:0.91; N = 121; P<0.0001). Flight speed varied significantly with both experiment number and forewing area, but was not affected by wing color or other variables tested here (Table S2).

To test whether associations between color and flight distance might be due to confounding effects of butterfly size [5], we used a general linear model to examine how wing hue varied with sex, experiment (categorical predictors) and forewing area (continuous covariate). Results showed significant effects of gender (F_1,114_ = 228.8, p<0.0001) and experiment (F_1,114_ = 49.2, p<0.0001) on wing hue, but no effect of forewing area (F_1,114_ = 2.39, p = 0.1246). We also tested for effects of monarch genetic relatedness on flight distance and wing hue using data from experiments 1 and 3 (each with 3 full-sib families represented; in experiment 2, all monarchs were full-siblings). Here we used a general linear model with experiment as a fixed factor, and family nested within experiment as a random effect. Results showed no significant differences in wing hue or flight distance across family lines (p>0.05 for both tests).

## Discussion

Our results show an association between monarch wing hue and flight endurance, with monarchs having the deepest-orange, or reddish-colored wings flying farther distances and for longer durations on average than those with yellower-colored wings. These experimental results are consistent with a prior study showing that wild monarchs captured during the fall migration in eastern N. America were on average redder than non-migratory individuals captured in the summer [5]. Because the monarch generation that emerges in late summer migrates to central Mexico and part-way back, long distance flight is of paramount importance to this cohort. It is possible that developmental pathways that affect flight endurance in this generation also influence wing pigmentation. Importantly, butterflies in our experiments were reared under similar environmental conditions: all larvae were fed greenhouse-grown cuttings of a single host plant species, *Asclepias incarnata*, and adults were flown under similar conditions of light and temperature (with minimal air currents). Thus, associations between flight performance and wing color observed here should reflect individual differences in these parameters and are unlikely to arise from external confounding variables.

To our knowledge, this study represents the first reported association of individual measures of flight performance and wing color (aside from melanism) within a single insect species. Most previous work on within-species variation in butterfly wing coloration has focused on the functional significance of melanism, for example, exploring the adaptive significance of and genetic basis for alpine melanism in relation to temperature limitations on flight [8], [18]. Other studies have examined pigments involved in mate choice, including the role of larval resource acquisition on the expression of male color traits [19], [20], the preference of females for melanic wing traits [21] and the role of UV pigments in butterfly mate choice [22]. In addition, a number of studies have examined seasonal forms of butterflies that vary in wing melanism and other morphometric traits, with darker forms predominantly found among generations experiencing cooler temperatures [7], [23], [24], [25].

Monarchs are known to vary in the degree of wing melanism among individuals [3], although our study showed no effect of wing melanism on flight endurance or speed. It is important to note that the thermoregulatory benefits of melanism might be expressed only under temperatures close to the flight threshold of butterflies [26]. Thus, flight muscles might warm up faster in darker individuals, allowing them to fly under colder temperatures [8], [18]. One explanation for the lack of an effect of melanism is that we tested monarch flight under relatively warm indoor conditions with exposure to only artificial fluorescent light, which might negate any potential thermoregulatory benefits arising from heat-absorptive properties of darker wings.

Further work is needed to uncover the mechanism causing the association between wing hue and monarch flight documented here. Correlations between these two variables could be caused by overlapping biochemical pathways during monarch development, or by metabolic constraints that affect both flight and wing color independently. We can surmise that variation in wing redness is caused by the differential deposition of color pigments such as pterins and ommochromes onto wing scales [27]. Indeed, microscopic comparisons of wings from monarchs with low to high hues showed that redder wings are associated with uniformly darker-pigmented scales, whereas yellower wings have scales with either uniformly light pigmentation, or with a mix of light and dark pigment (Fig. 4). Studies that compare the thoracic flight muscles, energy storage or metabolism across individuals with different hue scores could shed further light on this association [28]. Moreover, it would be important to determine whether color-flight associations are unique to migratory insects like monarchs, for which survival hinges on flight endurance, or also occur in other butterfly species. Finally, our results add new information to a growing body of work on the fitness correlates of wing color traits, expanding on their known roles as signals to potential mates and predators.

## Supporting Information

File S1 **Comparison of digital scan and reflectance spectra methods for quantifying wing color.**
(DOC)

File S2 **Summary tables of additional statistical analyses examining relationships between wing color and flight time and speed.**
(DOC)

Video S1 **Video of a monarch attached to the flight mill used in the experiments.**
(AVI)

## References

[1] Brower LP (1995). Understanding and misunderstanding the migration of the monarch butterfly (Nymphalidae) in North America: 1857–1995.. Journal of the Lepidopterists’ Society.

[2] Brower LP, Moffitt CM (1974). Palatability dynamics of cardenolides in monarch butterfly.. Nature.

[3] Davis AK, Farrey B, Altizer S (2005). Variation in thermally-induced melanism in monarch butterflies (Lepidoptera: Nymphalidae) from three North American populations.. Journal of Thermal Biology.

[4] Davis AK, Cope N, Smith A, Solensky MJ (2007). Wing color predicts future mating success in male monarch butterflies.. Annals of the Entomological Society of America.

[5] Davis AK (2009). Wing color of monarch butterflies (*Danaus plexippus*) in eastern North America across life stages: migrants are ‘redder’ than breeding and overwintering stages.. Psyche 2009.

[6] Fric Z, Konvicka M (2002). Generations of the polyphenic butterfly *Araschnia levana* differ in body design.. Evolutionary Ecology Research.

[7] Kingsolver JG (1995). Viability selection on seasonally polyphenic traits - wing melanin pattern in western white butterflies.. Evolution.

[8] Guppy CS (1986). The adaptive significance of alpine melanism in the butterfly Parnassius phoebus F (Lepidoptera: Papilionidae).. Oecologia.

[9] Ellers J, Boggs CL (2004). Functional ecological implications of intraspecific differences in wing melanization in *Colias* butterflies.. Biological Journal of the Linnean Society.

[10] Van Dyck H, Matthysen E, Dhondt AA (1997). The effect of wing colour on male bahavioral strategies in the speckled wood butterfly.. Animal Behavior.

[11] Altizer SM, Oberhauser K, Brower LP (2000). Associations between host migration and the prevalence of a protozoan parasite in natural populations of adult monarch butterflies.. Ecological Entomology.

[12] Bradley CA, Altizer S (2005). Parasites hinder monarch butterfly flight: implications for disease spread in migratory hosts.. Ecology Letters.

[13] Altizer S, Davis AK (2010). Populations of monarch butterflies with different migratory behaviors show divergence in wing morphology.. Evolution.

[14] Stavenga DG, Stowe S, Siebke K, Zeil J, Arikawa K (2004). Butterfly wing colours: scale beads make white pierid wings brighter.. Proceedings of the Royal Society of London Series B.

[15] Wilts BD, Pirih P, Stavenga DG (2011). Spectral reflectance properties of iridescent pierid butterfly wings.. Journal of Comparative Physiology A.

[16] Wijnen B, Leertouwer HL, Stavenga DG (2007). Colors and pterin pigmentation of pierid butterfly wings.. Journal of Insect Physiology.

[17] Statistica (2003). Statistica version 6.1, Statsoft Inc..

[18] Ellers J, Boggs CL (2002). The evolution of wing color in *Colias* butterflies: heritability, sex linkage, and population divergence.. Evolution.

[19] Morehouse NI, Rutowski RL (2010). Developmental responses to variable diet composition in a butterfly: the role of nitrogen, carbohydrates and genotype.. Oikos.

[20] Kemp DJ (2008). Resource-mediated condition dependence in sexually dichromatic butterfly wing coloration.. Evolution.

[21] Wiernasz DC (1989). Female choice and sexual selection of male wing melanin pattern in *Pieris occidentalis* (Lepidoptera).. Evolution.

[22] Morehouse NI, Rutowski RL (2010). In the eyes of the beholders: female choice and avian predation risk associated with an exaggerated male butterfly color.. American Naturalist.

[23] Berwaerts K, Matthysen E, Van Dyck H (2008). Take-off flight performance in the butterfly *Pararge aegeria* relative to sex and morphology: a quantitative genetic assessment.. Evolution.

[24] Van Dyck H, Wiklund C (2002). Seasonal butterfly design: morphological plasticity among three developmental pathways relative to sex, flight and thermoregulation.. Journal of Evolutionary Biology.

[25] Vandewoestijne S, Van Dyck H (2011). Flight morphology along a latitudinal gradient in a butterfly: do geographic clines differ between agricultural and woodland landscapes?. Ecography.

[26] Trullas SC, van Wyk JH, Spotila JR (2007). Thermal melanism in ectotherms.. Journal of Thermal Biology.

[27] Janssen JM, Monteiro A, Brakefield PM (2001). Correlations between scale structure and pigmentation in butterfly wings.. Evolution and Development.

[28] Marden JH, Cobb JR (2004). Territorial and mating success of dragonflies that vary in muscle power output and presence of gregarine gut parasites.. Animal Behaviour.

