# Supplementary material for: The Redder the Better: Wing Color Predicts Flight Performance in Monarch Butterflies
Source: PLoS One. 2012 Jul 25;7(7):e41323. doi: 10.1371/journal.pone.0041323 (PMC3405115; doi:10.1371/journal.pone.0041323)
Supplement: File S1 — Comparison of digital scan and reflectance spectra methods for quantifying wing color. (DOC) [file pone.0041323.s001.doc]

**Supporting Information File S1**

**Comparison of digital scan and reflectance spectra methods for quantifying wing color**

The method we used to measure wing color of monarchs based on scanned images differs from recent studies that use spectrophotometers to measure complete reflectance spectra of butterfly wings [1,2,3]. For this reason we compared the output of both methods using a set of captive raised monarchs (n=30, all males) that had recently eclosed in our lab. Three-day-old monarchs were chilled on ice for ease of handling, placed dorsal-side down on a flatbed scanner (Microtek ScanMaker i320) and held in place with small weights. The resulting scan (at 300dpi) was of a monarch in a standard pinning position (Fig. S1). During scanning, we ensured that the scanner settings were such that the image was not enhanced or adjusted in any way.

From the scanned images, we used image analysis software (FoveaPro, Reindeer Graphics, Inc.) for Adobe Photoshop ®, to measure the color of the central orange cell (cell CuA_1_, see Fig. S1) of the right and left forewing. For this we first selected the entire cell using the magic want tool (the area within the black vein) and ran a built-in color-measurement routine from FoveaPro, which returns the average pixel hue, saturation and brightness scores of the selected area. For most images at the resolution we used there were between 7000-9000 pixels. The hue score was the primary variable of interest for this study, measured in degrees (0-360°) with lower numbers indicating colors closer to red.

To measure spectral reflectance we used a USB2000 miniature fiber optic reflectance spectrophotometer from Ocean Optics (Dunedin, FL, USA), with an LS-1 Tungsten halogen light source, an R400-7-VIS ⁄ NIR reflection probe, and an RPH-1 probe holder from Ocean Optics. This model has a detection range of 200-1100 nm, and is the same model as that used in prior investigations of butterfly wing color [2,3]. Using the same set of monarchs that were scanned previously (n=30) we placed each dorsal-side up on a standard greycard and held the body in place with small weights. Next we placed the probe holder over the same wing cell measured previously (see Fig. S1) and took two readings of reflectance, which we averaged for each butterfly. Each reading produced one spectrum spanning 340–1026 nm and consisting of 2047 reflectance measurements. Reflectance readings for each wavelength were normalized relative to a diffuse reflection standard (model WS-1, Ocean Optics), which is uniformly reflective across 250–2000 nm.

RESULTS

Spectral curves from the CuA_1_ forewing cells of two monarchs (as measured from the spectrophotometer) that represented high and low hue scores (as measured by the scanner method) are shown in Figure S2. Reflectance values differed most notably between wavelengths of 580-780nm, for which reflectance values of the low-hue monarch were greater. Graphical inspection of data from other monarchs in this group showed similar patterns, with the visible among-individual variation occurring within this wavelength range.

For quantitative comparison of color data obtained from both methods we selected spectral readings within the range indicated by the dashed vertical lines in Fig. S2, starting at 580 nm, and at 20 nm intervals until 780 nm. These values were compared against the digital image hue scores for each butterfly using Pearson correlations. All comparisons (11 in total) showed significant correlations, with the highest p value (least significant correlation) being 0.011. The strongest relationship between hue and reflectance was at 640 nm (Fig. S3). All relationships were negative owing to the computer hue scoring system, where lower scores indicate colors closer to red. Similar comparisons between digital hue scores and wavelengths outside the range indicated in Fig. S2 showed no significant relationship (e.g., for 400nm, p=0.409 and for 900nm, p=0.399). Thus, this exercise demonstrated significant congruence between color variation captured by the scanner/image analysis and the spectrophotometer methods for measuring wing color of monarch butterflies between the visible color range of yellow (520nm) and red (780nm).

FIGURE LEGENDS

Figure S1. Scanned image of the dorsal side of a male monarch butterfly. Arrow points to cell CuA_1_ where color was measured using image analysis software and a spectrophotometer.

Figure S2. Reflectance spectra of two monarchs that vary in wing hue, as measured by the scanner method (blue line = hue score of 30, red line = hue score of 38). Dotted lines indicate wavelength range used for comparisons with the scanner method, and arrow indicates 640nm where the relationship between the data obtained by the scanner and spectrophotometer was strongest (see Fig. S3)

Figure S3. Relationship between the hue scores of 30 monarchs obtained using image analysis of scans and the spectral reflectance readings (at 640 nm) obtained using a spectrophotometer. Both measurements targeted the same wing cell (cell CuA_1_).

Figure S1


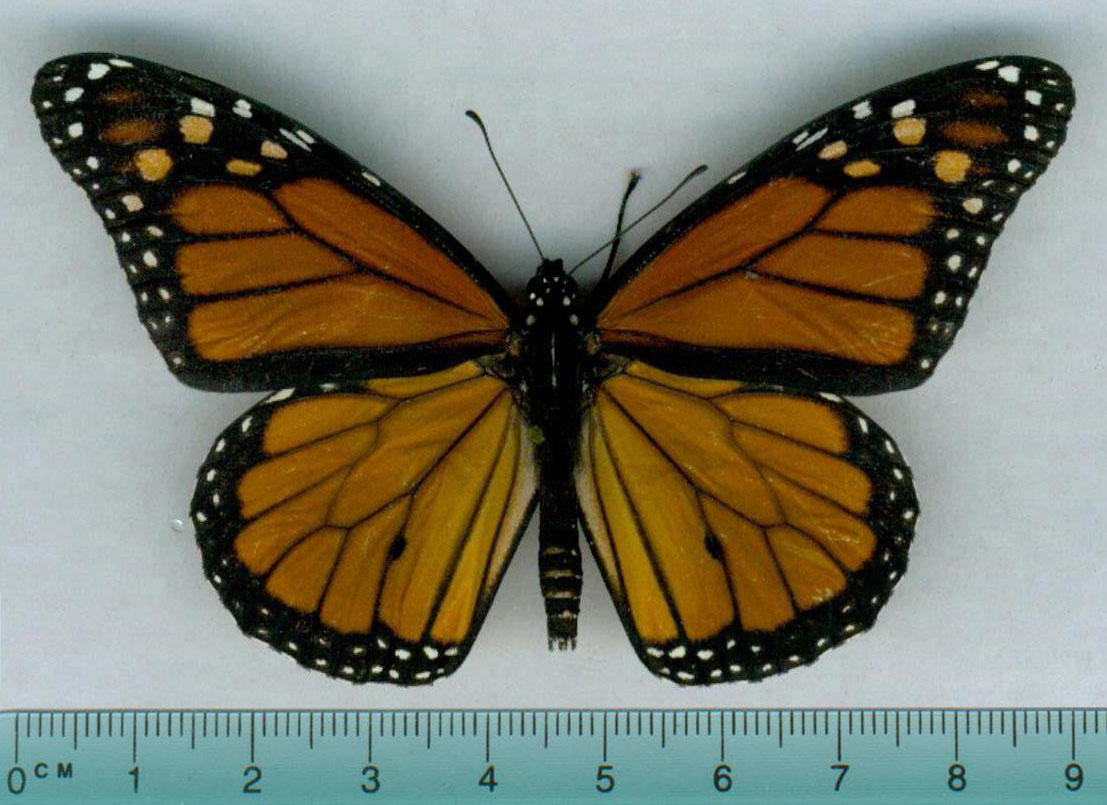


Figure S2

Figure S3


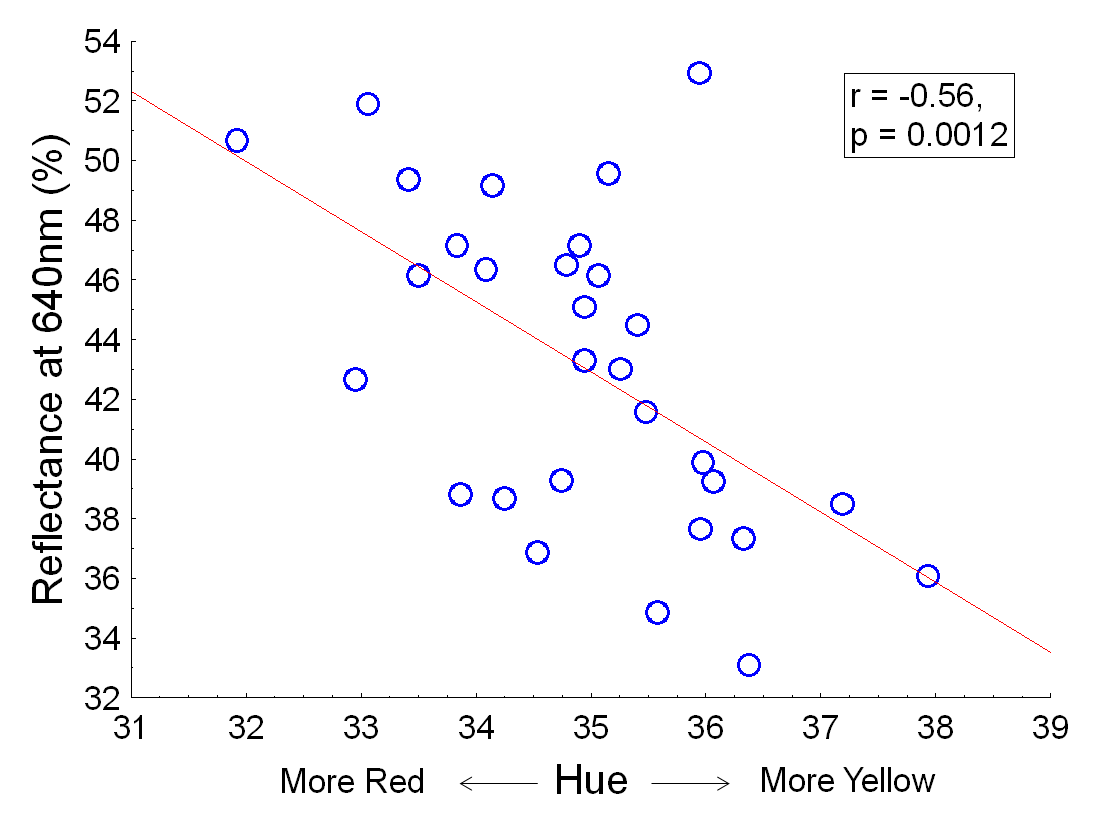


REFERENCES

1. Snell-Rood EC, Papaj DR (2009) Patterns of phenotypic plasticity in common and rare environments: a study of host use and color learning in the cabbage white butterfly *Pieris rapae*. American Naturalist 173: 615-631.

2. Morehouse NI, Vukusic P, Rutowski R (2007) Pterin pigment granules are responsible for both broadband light scattering and wavelength selective absorption in the wing scales of pierid butterflies. Proceedings of the Royal Society B-Biological Sciences 274: 359-366.

3. Friberg M, Vongvanich N, Borg-Karlson AK, Kemp DJ, Merilaita S, et al. (2008) Female mate choice determines reproductive isolation between sympatric butterflies. Behavioral Ecology and Sociobiology 62: 873-886.
