# Supplementary material for: The Redder the Better: Wing Color Predicts Flight Performance in Monarch Butterflies
Source: PLoS One. 2012 Jul 25;7(7):e41323. doi: 10.1371/journal.pone.0041323 (PMC3405115; doi:10.1371/journal.pone.0041323)
Supplement: File S2 — Summary tables of additional statistical analyses examining relationships between wing color and flight time and speed. (DOC) [file pone.0041323.s002.doc]

**Supporting Information File S2**

**Summary tables of additional statistical analyses examining relationships between wing color and flight time and speed**

Table S1. General linear model examining factors influencing total flight time (in minutes, square-root transformed) of monarchs across three flight mill experiments.

| **Variable** | **df** | **MS** | **F** | **p** |
| --- | --- | --- | --- | --- |
| **Sex** | 1 | 54.54 | 7.03 | 0.0092 |
| **Experiment** | 2 | 31.02 | 4.00 | 0.0210 |
| **Wing Hue (log)** | 1 | 96.99 | 12.50 | 0.0006 |
| **Relative Body Size** | 1 | 9.00 | 1.16 | 0.2836 |
| **Forewing Area** | 1 | 11.09 | 1.43 | 0.2343 |
| **Percent Black** | 1 | 6.89 | 0.89 | 0.3481 |
| **Aspect Ratio** | 1 | 5.31 | 0.68 | 0.4100 |
| **Error** | 112 | 7.76 |  |  |
| **Total** | 120 |  |  |  |

Table S2. General linear model examining factors influencing flight speed (km/hr) of monarchs across three flight mill experiments.

| **Variable** | **df** | **MS** | **F** | **p** |
| --- | --- | --- | --- | --- |
| **Sex** | 1 | 0.57 | 0.77 | 0.3816 |
| **Experiment** | 2 | 2.80 | 3.80 | 0.0252 |
| **Wing Hue (log)** | 1 | 0.36 | 0.50 | 0.4827 |
| **Relative Body Size** | 1 | 0.09 | 0.13 | 0.7208 |
| **Forewing Area** | 1 | 2.95 | 4.02 | 0.0474 |
| **Percent Black** | 1 | 0.17 | 0.23 | 0.6313 |
| **Aspect Ratio** | 1 | 0.03 | 0.04 | 0.8476 |
| **Error** | 112 | 0.74 |  |  |
| **Total** | 120 |  |  |  |
